# Molecular Interactions of Autophagy with the Immune System and Cancer

**DOI:** 10.3390/ijms18081694

**Published:** 2017-08-03

**Authors:** Yunho Jin, Yunkyung Hong, Chan Young Park, Yonggeun Hong

**Affiliations:** 1 Department of Rehabilitation Science, Graduate School of Inje University, Gimhae 50834, Korea; jynh33@naver.com; 2 Ubiquitous Healthcare & Anti-Aging Research Center (u-HARC), Inje University, Gimhae 50834, Korea; dangmoo777@naver.com; 3 Biohealth Products Research Center (BPRC), Inje University, Gimhae 50834, Korea; 4 Department of Physical Therapy, College of Biomedical Science & Engineering, Inje University, Gimhae 50834, Korea; 5 Department of Life Sciences, Ulsan National Institute of Science and Technology (UNIST), Ulsan 44919, Korea

**Keywords:** autophagy, immune system, cancer, cell death, metabolic homeostasis

## Abstract

Autophagy is a highly conserved catabolic mechanism that mediates the degradation of damaged cellular components by inducing their fusion with lysosomes. This process provides cells with an alternative source of energy for the synthesis of new proteins and the maintenance of metabolic homeostasis in stressful environments. Autophagy protects against cancer by mediating both innate and adaptive immune responses. Innate immune receptors and lymphocytes (T and B) are modulated by autophagy, which represent innate and adaptive immune responses, respectively. Numerous studies have demonstrated beneficial roles for autophagy induction as well as its suppression of cancer cells. Autophagy may induce either survival or death depending on the cell/tissue type. Radiation therapy is commonly used to treat cancer by inducing autophagy in human cancer cell lines. Additionally, melatonin appears to affect cancer cell death by regulating programmed cell death. In this review, we summarize the current understanding of autophagy and its regulation in cancer.

## 1. Introduction

The term autophagy is derived from the Greek word meaning “self-eating” [1]. Autophagy is a catabolic process in which intracellular components are sequestered and degraded for recycling [2]. This process occurs under conditions of amino acid starvation, glucose deprivation, oxygen deficiency, growth factor withdrawal, and cellular damage [3]. The degradation of damaged or long-lived proteins and organelles provides the cell with a new energy source for the recovery of homeostasis despite metabolic stress [3]. Three different types of autophagy have been identified: macroautophagy, microautophagy, and chaperone-mediated autophagy [1,4]. Macroautophagy results in the degradation of long-lived cytosolic proteins and organelles following their fusion with the lysosome and autophagosome, which engulfs the substrate [4]. In microautophagy, the substrates are directly engulfed by the vacuole membrane and subsequently degraded [5]. Microautophagy can be observed in some plant species during seed germination to degrade starch granules and storage proteins [6]. During chaperone-mediated autophagy, the target substrates are selected in a chaperone-dependent manner and translocated to the lysosome for degradation [7].

Among the three mechanisms, macroautophagy is the most common [1] and has received the most attention [5]. This review focuses on macroautophagy (referred to hereafter as autophagy) and its roles in the immune system and cancer. Since autophagy involves the transfer of cytoplasmic substrates to lysosomes, it has been implicated in both innate and adaptive immunities [8]. Innate immune receptors recognize pathogens, trigger the release of inflammatory cytokines, and induce pathogen removal through autophagy.

Defective autophagy and apoptosis may contribute to disease pathogenesis, including cancer [3,9], whereas the preservation of cellular homeostasis via autophagy is important for cancer prevention [10]. However, autophagy is a “double-edged sword”, because it not only suppresses but also promotes cancer cell survival [9,10]. These paradoxical functions of autophagy in cancer remain to be fully elucidated.

## 2. The Initiation of Autophagy during Cancer

**Figure 1:**
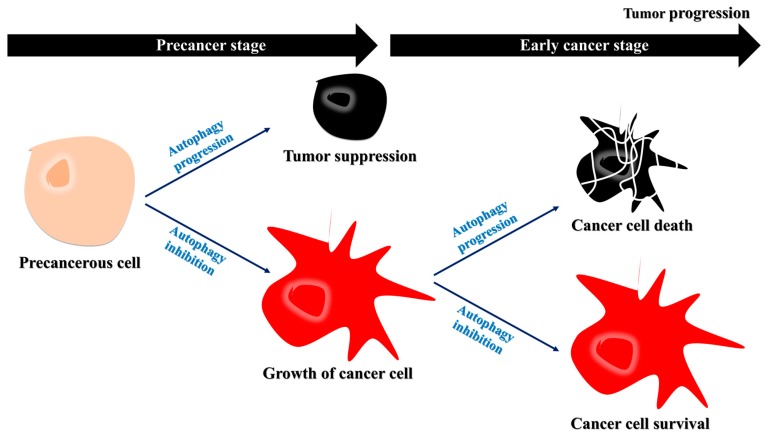
Decreased autophagy may favor cancer cell growth during the early stages of cancer progression. During the precancer stages, the inhibition of autophagy contributes to tumor growth. During the early stages of cancer, autophagy acts as a tumor suppressor, and the inhibition of autophagy can allow for the growth of cancer cells. Specifically, protein degradation by autophagy may interrupt tumor growth, suggesting that autophagy inhibition may be a therapeutic strategy for early cancer.

In cancer cells, autophagy is regulated by phosphatidylinositol 3-kinase (PI3K)/mammalian target of rapamycin (mTOR) and activated protein kinase (AMPK) pathways [11]. The activation of PI3K results in the production of phosphatidylinositol 3,4,5-trisphosphate (PtdIns(3,4,5)P3), which then binds to Akt [11], activating it and several downstream pathways, including mTOR [12]. AMPK is activated in response to energy depletion to induce autophagy [11]. Thus, autophagy inhibits cancer, whereas the inhibition of autophagy enables the growth of precancerous cells [12,13]. During the early stages of cancer cell development, protein synthesis rather than degradation is required for cancer cell growth [14,15]. Therefore, autophagy inhibition during these stages can cause cancer cell growth [15]. During the advanced stages of cancer, autophagy is upregulated because cancer cells exploit autophagy for their survival under starved conditions (Figure 1) [12,16]. Based on these observations, autophagy inhibition may be a therapeutic strategy for cancer during its early stages [15].

## 3. Autophagy as an Innate Immune Response against Cancer

Innate-immunity-mediated autophagy is regulated by the activation of innate immune receptors, including Toll-like receptors (TLRs) and nucleotide oligomerization domain (NOD)-like receptors (NLRs) [11]. TLRs induce inflammatory cytokine production by activating the NF-κB and mitogen-activated protein kinase (MAPK) pathways, mainly through myeloid differentiation primary response 88 (MYD88)-dependent pathways, either alone or in collaboration with Toll/interleukin-1 receptor (TIR) domain-containing adaptor molecule 1 (TICAM1)-dependent pathways [17,18,19]. TLRs are usually expressed in cancer cells, and are responsible for the regulation of autophagy as well as several immune responses [11]. Autophagy triggered by TLR3 and TLR4 was shown to contribute to the progression of lung cancer [17]. However, several studies have suggested that TLR activation enhances the survival, proliferation, and metastasis of cancer cells [17,20,21,22,23]. Furthermore, TLRs trigger the release of proinflammatory cytokines, chemokines, and immunosuppressive factors, leading to immune evasion and enhanced cancer cell resistance [17,23].

**Figure 2:**
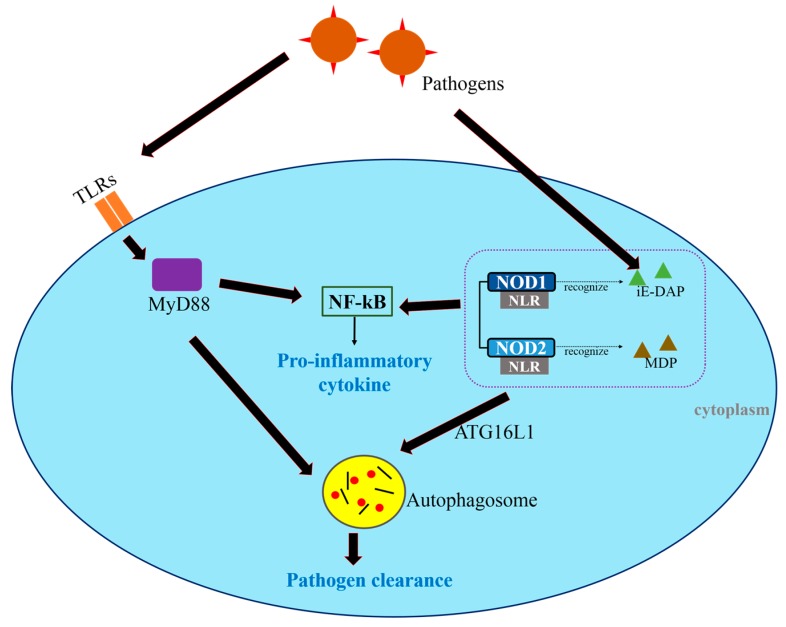
Toll-like receptors (TLRs) and Nod-like receptors (NLRs) regulate autophagy and the innate immune responses. Pathogens are identified by TLRs and NLRs. TLRs activate NF-κB through MyD88-dependent pathways, leading to the production of pro-inflammatory cytokines and the formation of autophagosomes that sequester intracellular p athogens. NLRs recognize cytosolic bacteria. The NLRs NOD1 and NOD2 detect γ-d-glutamyl-*meso*-diaminopimelic acid (iE-DAP) and muramyl dipeptide (MDP), respectively, and induce autophagosome formation. In this step, ATG16L1 facilitates bacterial trafficking. The pathogens are ultimately removed through autophagy.

While TLRs sense microbes on the cell surface, NLRs, which are important components of the innate immune system, recognize cytosolic bacteria. NOD1 and NOD2 detect intracellular microbes incorporating meso-diaminopimelic acid (iE-DAP) and muramyl dipeptide (MDP), respectively [24]. TLRs as well as NOD1 and NOD2 activate the NF-κB and MAPK pathways [24]. Moreover, they participate in regulating autophagy by interacting with ATG16L1, which mediates autophagosome formation and facilitates bacterial invasion through the cell membrane into the cells (Figure 2) [11,25,26]. NOD2 also recognizes invasive bacteria, thereby triggering autophagy and leading to NOD2-mediated host defenses [27]. Both NOD1 and NOD2 are thought to engage not only in innate and adaptive immune responses, but also in the interaction between autophagy and cancer [11,28]. By altering the balance between pro- and anti-inflammatory cytokines, NOD1 and NOD2 modulate the risk of cancer [28]. However, much remains to be learned about the contributions of TLR and NLR to cancer immunity.

## 4. Autophagy as an Adaptive Immune Response against Cancer

An adaptive immune response depends on the identification of extracellular or intracellular peptide epitopes presented by major histocompatibility complex (MHC) class II and I molecules, which are recognized by CD4^+^ and CD8^+^ T cells, respectively [29]. Antigen presentation is an important immune process accompanying the initiation of an adaptive immune response to protect organisms from pathogens. MHC II allows antigens to be identified by T cell receptors expressed by CD4^+^ T cells [30]. In addition, the antigen presentation step is known to be enhanced by autophagy through the acceleration of delivery of pathogen-related peptides to lysosomes [29,31]. Indeed, it has been reported that autophagy can affect γδ T cell-mediated interleukin (IL)-17, interferon (IFN)-γ, and IL-22 production in an IL-1 secretion-dependent manner, suggesting that autophagy is a pivotal regulator of immune responses [32]. Autophagy also modulates T and B lymphocytes, and plays a role in T cell survival, proliferation, homeostasis, and activation [11,33]. Basal autophagy is required for T cells to maintain homeostasis, and autophagy-related proteins are associated with T cell activation. Indeed, defective autophagy evoked by the deletion of pro-autophagic mediators such as *Atg3*, *Atg5*, *Atg7*, *BECN1*, and class III phosphoinositide 3-kinase/Vpas34 can disturb T cell homeostasis and survival [11]. Additionally, autophagy plays a role in B cell development and survival. Especially, Atg5 is essential for B cell development, and it may allow for a transition between pro- and pre-B cell stages in bone marrow [11].

The autophagy protein *Atg7* is required for T lymphocyte survival, and autophagy-deficient T cells exhibit increased reactive oxygen species generation, presumably due to the insufficient degradation of mitochondrial components [34]. Several studies have shown that defective autophagy induced by the ablation of pro-autophagic molecules, such as Vps34 and PI3K, is harmful to mitochondrial quality control, leading to a disruption of T cell homeostasis and survival [11,35,36]. In cancer, autophagy may induce not only the survival but also the death of T cells. In addition, autophagy may promote the helper T lymphocyte response, thus enhancing tumor recognition [37]. Alternatively, autophagy may provide cancer cells with a survival advantage, protecting them against immunosurveillance by suppressing CD4^+^ and CD8^+^ T cells [38]. Based on these observations, autophagy is thought to play a dominant role in T cell function.

Autophagy is needed for the survival and differentiation of B cells as well [39]. *Atg5*, an autophagy-related gene, contributes to B cell survival during development [40]. B cell activation is induced by tumor-derived autophagosomes (Dribbles), which sequester various tumor antigens in a TLR4/MYD88-dependent reaction [41]. However, autophagy has been explored less in B cells than in T cells.

## 5. Autophagy and Its Regulatory Function on Cancer Cell Fate

### 5.1. Autophagy Suppresses Tumor Development and Induces Cancer Cell Death

A relationship between disrupted autophagy and cancer development has been demonstrated. For example, *BECN1*, an autophagy-related gene required for autophagosome formation, seems to act as a tumor suppressor, and certain brain tumors have been attributed to insufficient *BECN1* expression [42]. A lack of the Beclin-1 protein was suggested to be involved in the malignant transformation of cells [43], and levels of the autophagy marker microtubule-associated protein 1 light chain 3 α (LC3) are reduced in cancer cells [44].

An interaction between autophagy and apoptosis to alleviate necrosis, leading to tumor suppression, has been proposed [45]. Autophagy may act as a tumor suppressor to limit tumor size [12]. These observations suggest that autophagy hinders tumor progression. Moreover, by sequestering damaged organelles, inducing cell differentiation, increasing protein catabolism, and promoting autophagic cell death, autophagy protects cells from becoming malignant [46]. Thus, while autophagy supports cell survival, it may also promote cell death in cases of imbalanced cell metabolism. Under the latter condition, autophagic cellular consumption surpasses the cellular capacity for protein synthesis [3]. However, autophagy may also protect cells from apoptotic cell death [15]. Furthermore, autophagy may induce cancer progression by increasing DNA damage and genomic instability [47].

### 5.2. Autophagy Drives Cancer Cell Survival

The well-known tumor suppressor protein, p53, can modulate autophagy. This protein has dual roles depending on the subcellular localization; p53 favors autophagy in the nucleus, whereas it suppresses autophagy in the cytoplasm. Under a normal environment, p53 levels are regulated by Mdm2-mediated ubiquitination. Under stressful conditions, p53 accumulates as a result of post-transcriptional modifications that allow it to avoid Mdm2-mediated degradation [48]. p53 in the nucleus then binds to several genes coding for pro-autophagic modulators [48]; p53 in the cytoplasm suppresses autophagy by inhibiting AMPK and activating mTOR [49].

**Figure 3:**
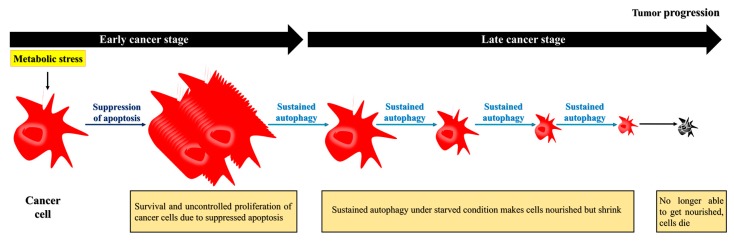
Apoptosis-defective cancer cells exploit autophagy to extend their survival, but eventually die. During the later stages, cancer cells require autophagy to survive under nutrient-deficient conditions. To survive under this environment, the cells may acquire nutrients recycled from damaged organelles through autophagy. Although the prolonged starvation can lead to cell death, cancer cells extend their life expectancy through autophagy.

Although the primary role of autophagy seems to prevent cancer, once a tumor develops, autophagy is exploited by cancer cells and has a protective role [9]. Indeed, cancer cells exploit autophagy to adapt to a stressful environment and maintain homeostasis in the presence of cellular stress. Cancer cells, especially their poorly vascularized internal regions, have been shown to utilize autophagy for survival under starvation and low-oxygen conditions [12,14]. Interestingly, autophagy-induced cancer cell survival is enhanced in cancer cells with defective apoptosis. Apoptotic cell death is suppressed in response to metabolic stress because of overexpression of the apoptotic inhibitor BCL2 [3]. The suppression of apoptosis prolongs cells survival, and may cause the uncontrolled proliferation of malignantly transformed cells that otherwise would have undergone apoptotic cell death [3,50]. These apoptosis-defective cancer cells undergo autophagy to extend their lifespan, as sustained autophagy not only nourishes the cells but also reduces their size, allowing them to survive under nutrient-poor/starvation conditions [3]. However, over time, the prolonged shrinkage and nutrient restoration can inhibit recovery, and, ultimately, induce cell death (Figure 3) [3]. Thus, while autophagy may induce cancer cell survival, as depicted in Figure 1, the cells eventually die due to sustained autophagy. In other words, both the survival and death of apoptosis-defective cancer cells under metabolic stress are dependent on autophagy.

### 5.3. Autophagy as a Candidate for Cancer Immunotherapy

Radiation therapy is the most commonly used treatment for cancer, as it possesses tumor growth-delaying properties [51,52]. Radiation induces autophagy in human cancer cell lines [52]. Autophagy following radiation therapy can have both cytoprotective and cytotoxic effects [52]. Autophagy induction can enhance the effect of radiation therapy in human oral squamous cancer cell by sensitizing the cells to irradiation. Additionally, the PI3K/Akt pathway (which is a major regulator of autophagy) is believed to regulate the cytotoxicity of post-radiation autophagy [52]. Conversely, other studies have shown that suppressing autophagy may augment radio-sensitization in human glioma cells [52,53]. Therefore, Janus-faced autophagy may induce either survival or death depending on the cell/tissue type [52].

Anti-cancer therapies can induce autophagy in apoptosis-defective cells, as the resultant cytotoxicity may evoke progressive autophagy, leading to autophagic cell death [3]. Therefore, the therapeutic induction of autophagy may contribute to cancer cell removal. As described above, cells defective in apoptosis undergo autophagic death. This property has been exploited in the development of anti-cancer therapies, which cause cells to become resistant to apoptosis [54]. Thus, promoting autophagic death in cancer cells can be a therapeutic approach for cancer treatment [54].

Generally, harmful stimuli induce apoptosis and autophagy during the early stages of cancer [55]. In a previous report, we discussed the therapeutic potential of the chronobiological hormone, melatonin, against colon cancer [56]. It has been reported that impaired melatonin release may be observed in patients suffering from colorectal cancer [56,57], and this cancer is prevalent in night-shift workers, suggestive of an association between melatonin and oncogenesis [56,58]. According to our previous study, melatonin increased HCT116 colorectal cancer cell death during the early stages by activating apoptosis and autophagy, which is supported based on the upregulation of bax, cleaved caspase 3, Beclin1, and LC3, as well as decreased AKT/pAKT expression [56]. Moreover, we observed a growth inhibition of cancer cells mediated by melatonin receptors. We also observed time-dependent MT1 downregulation and early upregulation of RORα in cancer cells. As the membrane melatonin receptor, MT1 may have an antiproliferative effect and is known to be overexpressed in malignant cells; melatonin-mediated anticancer effects may cause MT1 downregulation in a time-dependent manner. RORα, which is a type of nuclear melatonin receptor, mediates immune function. Therefore, early upregulation of RORα is believed to be due to the early tumor suppressive effect of melatonin through its nuclear receptors [56].

High-mobility group box 1 protein (HMGB1) is a nuclear protein that functions as a transcriptional enhancer and mediates inflammatory responses [59]. HMGB1 is secreted from inflammatory cells to evoke inflammation by binding to receptors such as the receptor for advanced glycation end products (RAGE) and TLR2/4 [55]. HMGB1 release is involved in the immune response against cancer cells [60]. This protein is not released from apoptotic cancer cells, indicating that apoptotic cell death is non-inflammatory [61]. HMGB1 has also been shown to inhibit apoptosis while promoting autophagy [55]. Reduced HMGB1 expression may increase cancer cell sensitivity by limiting autophagy [55]. Based on the association between autophagy and apoptosis in cancer cells, HMGB1 may be an important therapeutic target in cancer treatment.

Recently, autophagy has been proposed to regulate the stemness of cancer stem cells (CSCs) [62]. Since CSCs can regenerate and differentiate, they are considered a major hurdle in the development of anticancer therapies. The stemness of CSCs can be increased through the eliminating and/or recycling properties of autophagy [62]. The optimal regulation of autophagy for improved cancer treatment has not been thoroughly characterized; however, CSC-stemness modulation through autophagy may be a potential therapeutic strategy.

## 6. Conclusions

As discussed above, inhibiting autophagy increases cancer cell growth; thus, promoting autophagy may suppress cancer during its early stages. In late-stage cancer, cancer cells use autophagy to protect themselves from anti-cancer therapy. Therefore, autophagy inhibitors (e.g., bafilomycin A1) can evoke cancer cell death through apoptosis and act as a cancer suppressor during these stages. In other words, autophagy limits cancer cell development and promotes the survival of existing cancer cells. Since cancer cells primarily undergo autophagy for self-protection, the inhibition of autophagy has generally been proposed as a therapeutic strategy against cancer. However, the promotion of autophagy may result in anti-cancer activity by inducing cytotoxic immune cells. Indeed, within the same cancer, both inhibition and promotion of autophagy may be beneficial. Therefore, an optimal combination of autophagy inhibition and promotion, according to the properties of the cancer, is needed. A better understanding of the roles of autophagy in cancer and immunity, and whether its induction or suppression will provide the desired effect, requires further study.
